# Supplemental oxygen induced cardiovascular pathophysiology in an end‐of‐life care model in mice

**DOI:** 10.14814/phy2.71045

**Published:** 2026-08-07

**Authors:** Rohan Cheruku, Drithi Chidanand, Sashank Rangarajan, Nishank Rangarajan, Siva Kumar Panguluri

**Affiliations:** ^1^ Department of Pharmaceutical Sciences Taneja College of Pharmacy, University of South Florida Tampa Florida USA; ^2^ Cell Biology, Microbiology and Molecular Biology College of Arts and Sciences, University of South Florida Tampa Florida USA

**Keywords:** cardiac remodeling, electrical remodeling, end‐of‐life care, sex disparities, supplemental oxygen

## Abstract

Supplemental oxygen in critical care patients is associated with increased mortality. We previously reported hyperoxia‐induces cardiac pathophysiology influenced by age, metabolic status, sex, and duration of exposure. The present study investigated the dose‐dependent effects of oxygen on cardiac remodeling in very aged mice (>100 weeks), a model relevant to end‐of‐life physiology and clinical decision‐making. Mice were exposed to graded oxygen concentrations or normal air for 72 h followed by comprehensive assessment of cardiac function and electrophysiology. Exposure to 70% O_2_ led to significant reduction in ejection fraction (82.5% ± 2.1% to 59.8% ± 3.4%) and cardiac output (38.2 ± 1.5 mL/min to 12.8 ± 1.2 mL/min in males and 8.1 ± 0.9 mL/min in females). A significant electrical remodeling (QTc 57.2 ± 1.8 ms to 78.4 ± 2.5 ms) was also observed. Although females exhibited higher baseline cardiac function than males, they demonstrated greater functional decline and earlier onset of electrical abnormalities at lower oxygen concentrations. These findings indicate that oxygen concentration is a critical determinant of cardiac dysfunction in advanced age, with heightened susceptibility in females. Collectively, this study highlights the potential risks of supplemental oxygen and underscores the importance of precision oxygen therapy in end‐of‐life care settings.

## 1 INTRODUCTION

Oxygen supplementation in the form of mechanical ventilation (MV) is one of the most frequently administered therapies for cardiac ICU patients, particularly in those suffering from acute respiratory distress. Short‐term MV is associated with increased survival in patients suffering from respiratory distress associated with acute cardiovascular conditions such as myocardial infarction, cardiac arrest, or pulmonary edema (Dias‐Freitas et al., 2016), while long‐term MV can lead to hyperoxia and thus is associated with elevated mortality. The mean duration of MV in ICU patients is 3 days (Lee & Cho, 2020); however, longer periods of MV up to 21 days have been used during the COVID‐19 pandemic, particularly in patients with COVID‐19 associated lung injury and pneumonia (Butler et al., 2023).

Supplemental oxygen and mechanical ventilation are common among older patients. In a Medicare‐based study of people with Chronic Obstructive Pulmonary Disease (COPD), about 25.9% of beneficiaries received supplemental oxygen in 2024 (Duan et al., 2024). Home oxygen also has ongoing monthly costs as recent analyses of claims data report median monthly payments for oxygen supplies in the ballpark of about $60–$66 per month (IQRs reported), and home concentrators or rental programs can add both equipment and electricity costs to the household and health‐system bill, with costs only increasing with time (Clark et al., 2024). For mechanically ventilated very elderly patients, outcomes are poor: end‐of‐life cohorts show very high 30‐day and 1‐year mortality. For example, one study reported ~72% 1‐year mortality among patients who received invasive ventilation, underscoring the clinical and economic burden of invasive respiratory support at the end‐of‐life (Leclaire et al., 2025).

Dyspnea is one of the most common and distressing symptoms experienced by patients at the end‐of‐life, affecting up to 70%–90% of individuals with advanced cancer, heart failure, or chronic lung disease (Abernethy et al., 2010; Currow et al., 2010; Fardy, 2016). Relief of dyspnea is a central goal of palliative and hospice care, yet treatment strategies remain largely empirical. Supplemental oxygen is among the most frequently prescribed interventions, even for patients without documented hypoxemia (Fardy, 2016; Uronis et al., 2008). Despite the frequency at which oxygen is administered to both ICU and elderly patients in hospice or palliative care, there is no clear set of guidelines to define when a patient needs to be prescribed oxygen. Several studies have found that oxygen used to treat patients after COPD hospitalization is potentially unneeded (Esteban et al., 2002; Spece et al., 2021). These meta‐analyses show the degree to which oxygen is used as a blanket treatment, with Medicare and Medicaid Services reporting that at least 1 million people used supplemental oxygen in 2021. These facts raise important questions on whether supplemental oxygen is prescribed to alleviate psychological assurance and a feeling of comfort for both the patient's family and medical staff, and the need for clearer information on the potential detrimental effects for the elderly. Additionally, randomized clinical trials have shown inconsistent or minimal benefit of oxygen therapy for dyspnea in non‐hypoxemic end‐of‐life patients (Abernethy et al., 2010; Ekström et al., 2016).

In previous studies, our lab has used a murine model to examine structural and functional cardiac remodeling events such as the development of arrhythmias, injury to cardiac tissue, QT prolongation, and decreases in CO and SV in a time, age, and sex‐dependent manner (Ayalasomayajula et al., 2024; Bojkovic et al., 2021; Chapalamadugu et al., 2015; Rodgers et al., 2018; Rodgers et al., 2021; Rodgers, Iyer, et al., 2019; Rodgers, Rodgers, et al., 2019; Saleem et al., 2023; Vichare et al., 2022). Our most recent study examined the impact that length of exposure to hyperoxia has on cardiac pathophysiology in young (~8–10 weeks old) and aged (~72 weeks old) mice of both male and female sexes, where we found that 72 hours of hyperoxia was sufficient in causing significant weight loss, electrophysiological remodeling events, and lung edema in all age and sex groups (Vichare et al., 2022). While prior studies have indicated the progression of hyperoxia‐induced cardiac pathophysiology in a time‐dependent manner, along with age and sex as additional risk factors, this is the first study investigating the impact of supplemental oxygen on cardiac pathophysiology in the context of quality of life and continuity of care in end‐of‐life care.

Mortality following MV in critical care units remains significantly higher in patients aged 70 years than the rest of the population regardless of sex (Esteban et al., 2002). The physiological impact of hyperoxia is driven by increased oxidative stress, which reduces coronary blood flow and promotes cardiomyocyte growth via redox‐sensitive signaling pathways (Farquhar et al., 2009; Giordano, 2005). We hypothesized that supplemental oxygen induces dose‐dependent cardiac and electrical remodeling in end‐of‐life mice, even at concentrations traditionally considered sub‐hyperoxia.

## 2 MATERIALS AND METHODS

### 2.1 Animals

All experiments were conducted using aged (>100 weeks or 24 months old) C57BL/6 male (*n* = 37) and female (*n* = 36) mice. Aged mice were obtained from the National Institute of Aging (NIA) housing facility and Jackson Laboratories (Chicago, IL). The mice were divided into three treatment groups (*n* = 20) based on O_2_ concentration (40% O_2_, 60% O_2_, and 70% O_2_) and were further split into male (*n* = 10) and female blocks (*n* = 10). We chose these FiO_2_ concentrations based on clinical settings in end‐of‐life or palliative care where supplemental oxygen is administered via nasal cannula (deliver up to 40% FiO_2_), simple face mask (up to 60%), and nonrebreather mask (up to 100%). Mice receiving room air (21% O_2_) were used as a control group (male *n* = 7, and female *n* = 6). To predict the number of animals to be used in each group, we used a sample size calculator (http://www.biomath.info/power/prt.htm) and calculated the sample size based on our experimental groups. Throughout the experiment, all animals were given access to food and water ad libitum and were housed in a room with a timed 12‐h light/dark cycle. All mice were weighed before and after each O_2_ treatment.

All animal experiments were strictly monitored and approved by the Institutional Animal Care and Use Committee (IACUC) at the University of South Florida, in compliance with guidelines set by the National Institutes of Health (NIH). Additionally, this study was conducted in accordance with the ARRIVE (Animal Research: Reporting of In Vivo Experiments) guidelines. Mice were randomly assigned to treatment groups, and all subsequent echocardiographic and electrocardiographic data analyses were performed by investigators blinded to the treatment assignments to ensure objectivity.

### 2.2 Hyperoxia exposure

Mice from each of the three treatment groups (40% O_2_, 60% O_2_, and 70% O_2_) were housed in an airtight chamber with a size of 50 × 50 × 30 cm for 72 h. During hyperoxia exposure, oxygen concentrations were controlled via an oxygen monitor (Vascular Technology, Chelmsford, MA) set to each respective treatment group's O_2_ concentration. Control group mice were housed with standard room air.

After post‐exposure 2D‐echocardiograms and surface ECGs were taken, and the mice received 180 USP units/100 μL heparin followed by IP 50 mg/kg euthasol to euthanize for thoracotomy. Hearts and lungs were dissected and stored at −80°C for further analysis.

### 2.3 Physical parameters

Assessment of the body weights of male and female mice was performed before and immediately after oxygen exposure. The changes in the body weights were recorded in groups of both sexes. The body weights were normalized with the tibia length. Lung wet to dry weight ratio was also recorded in all groups to assess supplemental oxygen‐induced lung edema as described in our previous publications (Rodgers et al., 2021).

### 2.4 Electrocardiography

Mice were anesthetized using ~2% isoflurane prior to the insertion of electrical leads, placed in the Lead II configuration as done in past studies (Chapalamadugu et al., 2015). For each mouse, we recorded a minimum of six 30 second ECG intervals using LabChart Pro software (AD Instruments), which we also utilized for subsequent analysis of our recordings. During analysis of ECG recordings, we measured RR, PR, JT, and QRS intervals. QT intervals were measured from the start of the Q wave up to the return of the T wave to the isoelectric baseline; Bazett's formula $\left(QT_{c}=\frac{\mathrm{QT}}{\sqrt{\mathrm{RR}}}\right)$ was used for the calculation of heart‐rate corrected QT interval (QTc) length.

All measurements were taken in milliseconds (ms).

### 2.5 Echocardiography

For transthoracic echocardiography, all recordings were taken using a Vevo3100LT Ultrasonograph equipped with a 30 MHz transducer (VisualSonics). Using echocardiography, we observed live cardiac function on all mice before and after oxygen treatments, regardless of O_2_ concentration. While taking recordings, mice were anesthetized with ~2% isoflurane and maintained at a 37°C body temperature using a heating pad. Two‐dimensional (2D) M‐mode recordings of the parasternal long and short axes at the midpapillary muscle were taken. On the parasternal short axis view, recordings across both the anterior and posterior walls were taken. From these recordings, left ventricular internal diameter (LVID), left ventricular anterior wall (LVAW), and left ventricular posterior wall (LVPW) were measured during systole (s) and diastole (d) using Vevo Lab software (VisualSonics). Ejection fraction (EF%) was calculated as: %EF = ((EDV‐ESV)/EDV) x 100 where EDV is end‐diastolic volume and ESV is end‐systolic volume. Stroke volume (SV, mL) was calculated as EDV – ESV. Cardiac output (CO, mL/min) was calculated as SV × HR, with HR taken from ECG readings. To ensure functional assessments were not confounded by anesthesia, average heart rates were maintained at a stable range (450–550 bpm) across all experimental groups during recording.

### 2.6 Statistical analysis

A mixed‐model ANOVA was used to estimate how the measured parameters were influenced by oxygen concentration and sex, with *p* < 0.05 representing statistical significance. Multiple comparisons were done using a Tukey Honest Significant Difference test. All tests were performed using the GraphPad Prism program. Analysis assumed that the data had equal variance and a normal distribution. Error bars represent ± SEM (Standard Error of the Mean).

## 3 RESULTS

### 3.1 Effect of oxygen concentrations on cardiac remodeling physical parameters

**FIGURE 1:**
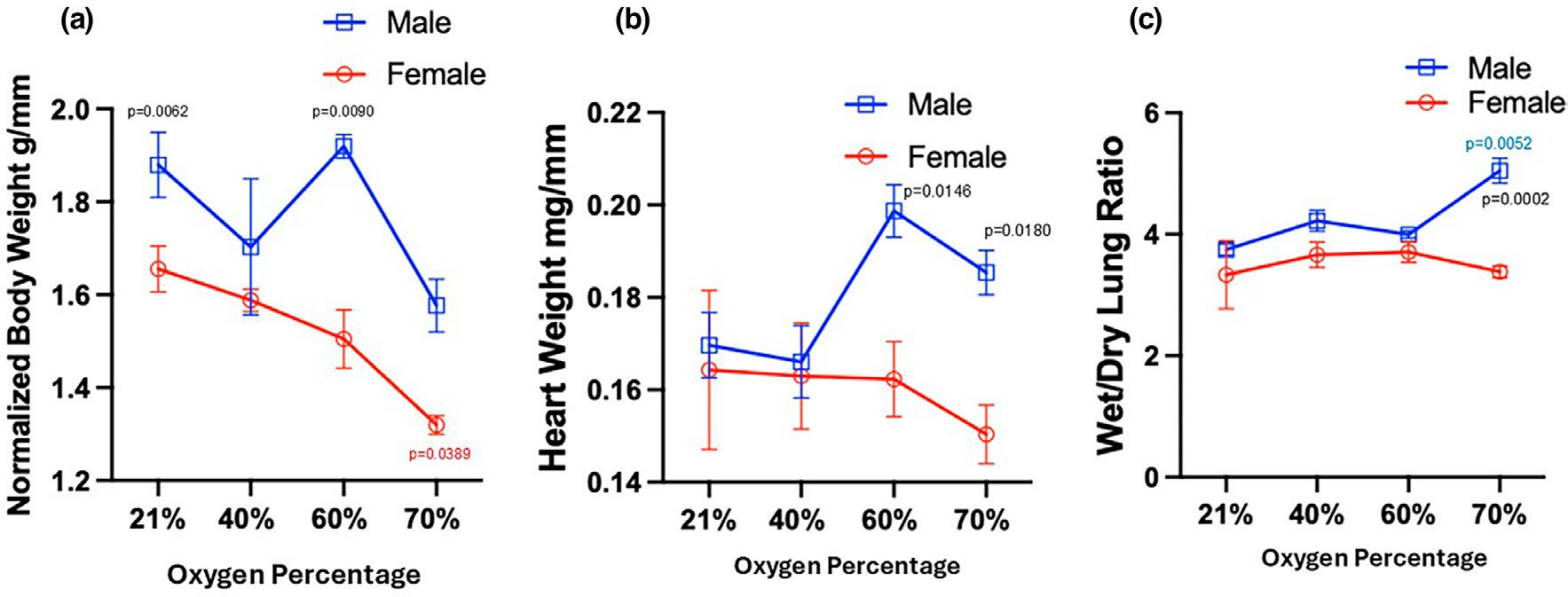
Impact of supplemental oxygen on cardiac physical parameters: Body weights (a), heart weights (b) normalized to tibia lengths, and lung wet to dry weight ratio (c) were plotted for all groups. Graphs shown here are mean (±SE). Here *p*‐value between normal air and various supplemental oxygen concentrations are presented in red (female) or blue (male), whereas *p*‐values between male and female groups were presented in black font.

When body weights were normalized with tibia lengths and compared to normal air (21% O_2_) group, none of the groups in male showed significance despite a clear trend of declining body weights (Figure 1a). Whereas in female group, there is significant decrease in body weights in 70% O_2_ group compared to their normal air controls (Figure 1a). On the other hand, male mice showed significant increase in heart weights starting from 60% O_2_ concentrations, leaving no significant change in female group compared to their normal air controls (Figure 1b). Lung wet to dry weight rations in female does not show any significant difference compared to their normal air control, indicating lack of any signs of lung edema, whereas male group showed significant lung edema after 70% O_2_ treatment (Figure 1c).

### 3.2 Functional parameters

**FIGURE 2:**
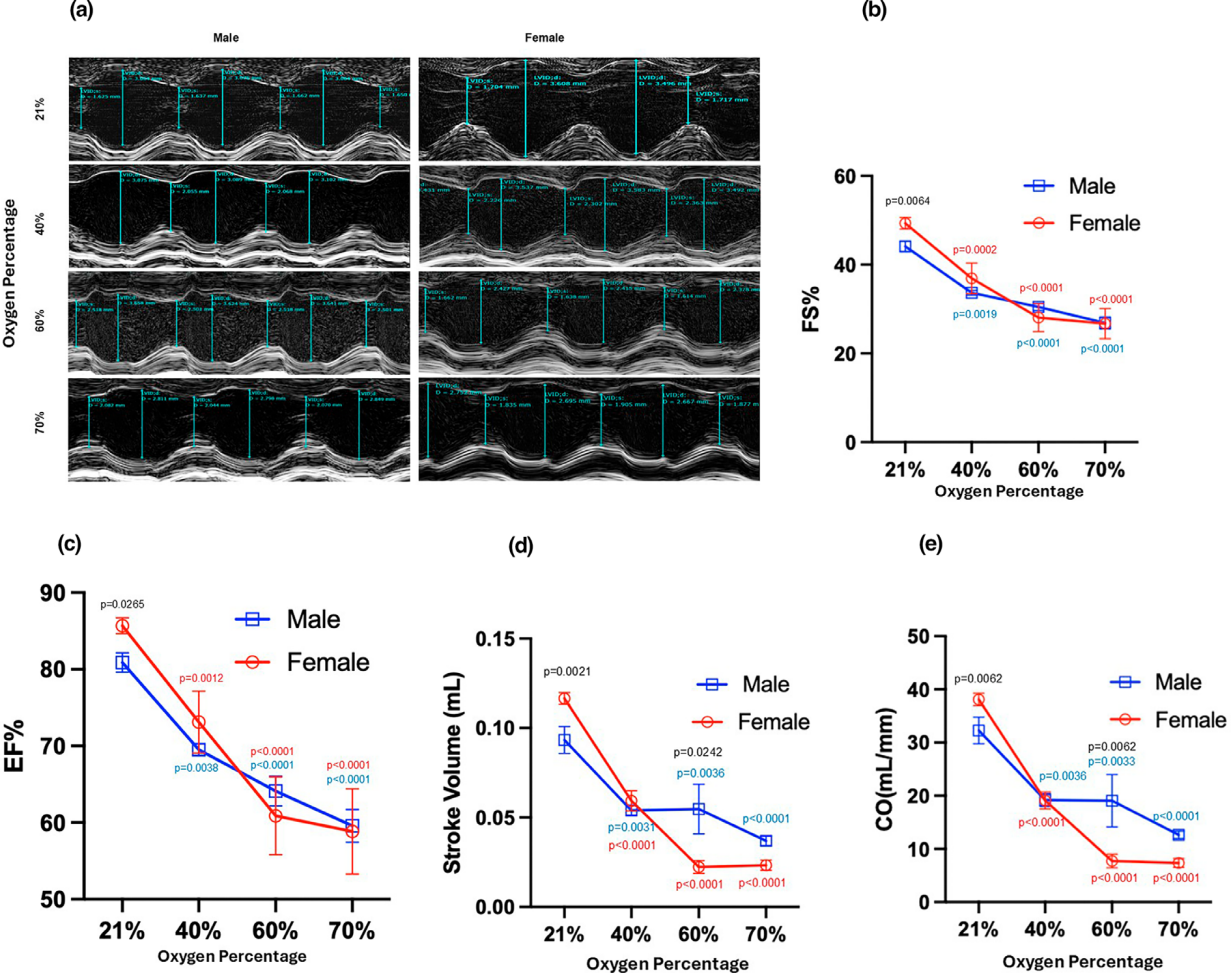
Functional abnormalities with supplemental oxygen in end‐of‐life mouse hearts: 2D‐echocardiogram images in short‐axis mode (a), %FS (b), %EF (c), stroke volume (d), and cardiac output (e) from all groups. Graphs shown here are mean (±SE). Here *p*‐value between normal air and various supplemental oxygen concentrations are presented in red (female) or blue (male), whereas *p*‐values between male and female groups were presented in black font.

Functional parameters using 2D echocardiogram showed a clear and significant reduction with increasing oxygen concentrations (Figure 2a–e). Both sexes showed a significant decrease in %FS in all oxygen treatment groups compared to their normal air controls (Figure 2b). Similarly, ejection fraction significantly decreased in both sexes starting from 40% O_2_ treatment group, which continues to decrease with higher O_2_ concentrations (Figure 2c). Stroke volume (Figure 2d) and cardiac output (Figure 2e) also showed significant decrease in both sexes even at low O_2_ concentrations compared to their normal air controls, which further worsen with increasing O_2_ concentrations.

### 3.3 Electrophysiological parameters

**FIGURE 3:**
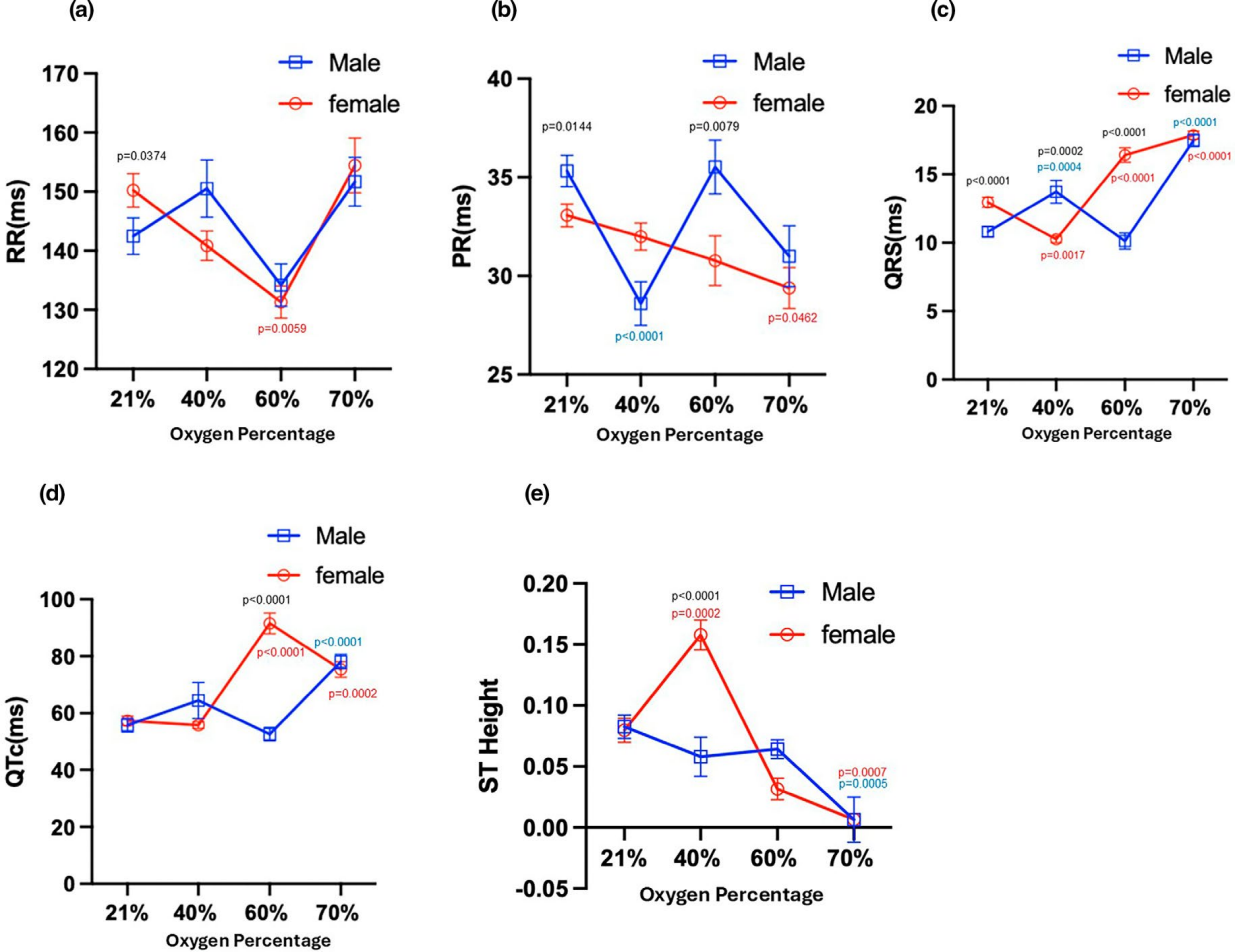
Electrical remodeling induced by supplemental oxygen in end‐of‐life mouse model: Surface ECG in lead II mode were taken and data was plotted for RR (a), PR (b), QRS (c), QTc (d) intervals, and ST height (e) from all groups. Graphs shown here are mean (±SE). Here *p*‐value between normal air and various supplemental oxygen concentrations are presented in red (female) or blue (male), whereas *p*‐values between male and female groups were presented in black font.

Exposure to increasing oxygen concentrations produced clear alterations in multiple electrical parameters when compared with normal air controls (Figure 3a–f). RR interval demonstrated a non‐linear pattern, decreasing from the baseline at normal air to significantly lower values at 60% O_2_, followed by a marked increase at 70% O_2_ in both sexes (Figure 3a). PR interval in males also varied nonlinearly with oxygen concentration, with the only significant difference from normal air being the 40% O_2_ treatment (Figure 3b). In females, the PR interval showed a consistent incremental decrease in females which did not reach statistical significance until at 70% O_2_ (Figure 3b). QRS duration increased significantly in male group only at 40% and 70% O_2_ treatments, whereas initial decline significantly at 40% following steep increase at 60% and 70% O_2_ treatments in female group compared to their normal air controls (Figure 3c). Similarly, Female mice showed significant increase in QTc interval starting from 60% O_2_, whereas no significant change until 70% O_2_ in males (Figure 3d). Although ST heights are consistently decreased in male groups with increased O_2_ concentrations, but they were not significant until 70% O_2_ (Figure 3e). Whereas in female group, ST heights were significantly elevated at 40% O_2_ while significantly reduced ST heights at 70% O_2_ concentrations compared to their normal air controls (Figure 3e). Taken together, the data demonstrates distinct oxygen‐dependent shifts across multiple electrical indices associated with cardiac electrical remodeling.

### 3.4 Sex influence on oxygen induced cardiac remodeling physical parameters

Male groups showed significantly higher body weights across all oxygen concentrations except 40% O_2_ compared to their female counterparts (Figure 1a). When we compared heart weights, male hearts are significantly higher than female counterparts only at 60% and 70% O_2_ treated group without any significant difference at normal air and 40% O_2_ (Figure 1b). No significant difference between male and female groups in lung wet to dry weight ration except at 70% O_2_ group (Figure 1c).

### 3.5 Functional parameters

Female group displayed significantly better %FS and %EF compared to their male counterparts at only at norma air with no significant differences in higher O_2_ concentrations (Figure 2b,c). Although female group showed significantly higher SV and CO at normal air; however, these values were significantly lower after 60% and 70% O_2_ treatments compared to their male counterparts (Figure 2d,e).

### 3.6 Electrophysiological parameters

Sex‐specific patterns were evident across electrical parameters in response to supplemental oxygen. Female group showed significantly high heart rates than males at normal air, with no difference in all other higher O_2_ treatments (Figure 3a). On the other hand, male group showed significantly higher PR intervals at normal air and after 40% O_2_ treatment, without any differences at 60% and 70% O_2_ compared to their female counterparts (Figure 3b). Female groups showed significantly higher QRS intervals at normal air later which significantly dropped down at 40% O_2_ and back to significantly higher QRS intervals than males at 60% O_2_ indicating a dynamic change with varying O_2_ concentrations (Figure 3c). Surprisingly female group did not show any difference in QTc interval at normal air compared to their male counterparts, except significantly higher QTc intervals at 60% O_2_ (Figure 3d). Similarly, no significant differences were observed with ST heights between male and female except at 40% O_2_, where females had significantly higher ST heights than males (Figure 3e).

## 4 DISCUSSION

Supplemental oxygen is routinely administered in end‐of‐life care despite limited evidence defining concentration‐dependent benefit or harm. Clinical studies indicate that oxygen therapy does not consistently relieve dyspnea in non‐hypoxemic patients (Abernethy et al., 2010) and that excessive oxygen exposure can impair cardiovascular function and increase adverse outcomes (Farquhar et al., 2009; Girardis et al., 2016). Our lab is the first to report excessive oxygen (specifically hyperoxia) as an independent risk factor for cardiovascular disease (CVD) using mouse model with a logical and systematic approach (Chapalamadugu et al., 2015; Panguluri, Tur, Chapalamadugu, et al., 2013; Panguluri, Tur, Fukumoto, et al., 2013). Although we established hyperoxia‐induced cardia pathophysiology in mouse model (Ayalasomayajula et al., 2024; Chapalamadugu et al., 2015; Panguluri, Tur, Fukumoto, et al., 2013; Rodgers et al., 2018; Rodgers, Rodgers, et al., 2019; Vichare et al., 2022; Vysotskaya et al., 2018), this is the first report investigating cardiac physiology in mouse model subjected to O_2_ concentrations lower than hyperoxia. Also, this is the first report investigating effect of supplemental oxygen on cardiac physiology in end‐of‐life mouse model. Clinical oxygen therapy often focuses on maintaining peripheral saturation (SpO_2_), but our findings suggest that the heart possesses an independent sensitivity to oxygen concentrations as low as 40%. This highlights a potential cardiovascular risk in end‐of‐life patients that may not be detected through standard respiratory monitoring alone. Undeniably, these findings question the notion that supplemental oxygen is always safer. Similar to any pharmacological agent, oxygen must be administered with caution, adjusted to the minimum effective amount, and continuously observed for possible cardiac adverse effects.

### 4.1 Physical parameters

Significant reduction of body weight after hyperoxia exposure has already been reported in our previous studies in both sexes and all ages (Ayalasomayajula et al., 2024; Bojkovic et al., 2021; Panguluri, Tur, Fukumoto, et al., 2013; Rodgers et al., 2021; Rodgers, Rodgers, et al., 2019; Saleem et al., 2023; Vichare et al., 2022). Reports also suggest weight loss in long‐term mechanically ventilated patients due to swallowing impairment (Ambrosino & Clini, 2004; Willig et al., 1996). Additionally, data from our previous study suggest reduced water intake in hyperoxia treated mice, at least in part, causing significant weight loss (Rodgers et al., 2021). When we initially exposed end‐of‐life mice to hyperoxia (FiO_2_ ≥90%), none of the mice survived beyond 24 h; therefore, we limited our experiments to the maximum FiO_2_ of 70%. Surprisingly, we do not observe any significant weight loss in the male group, whereas significant weight loss occurred only after 70% O_2_ treatment in female mice (Figure 1a). Similar to young (Rodgers, Rodgers, et al., 2019) and aged (Vichare et al., 2022) mice groups, end‐of‐life female mice also showed significantly smaller body sizes in almost all treatment groups compared to their male counterparts (Figure 1a). Although the exact reason and mechanism are not known, reports suggest that women show three times more muscular atrophy than men in ICU (Wu et al., 2022). Therefore, it is evident that supplemental oxygen can cause significant reduction of body weights in female end‐of‐life mice at higher O_2_ concentrations, which may eventually reduce quality of life and comfort.

Unlike body weights, heart weights in end‐of‐life male groups not only showed a significant increase after 60% O_2_ treatment but also were significantly heavier than their female counterparts, while there was no significant change in female hearts across all O_2_ treatments (Figure 1b). Although there are no reports on the impact of supplemental oxygen on heart weight at concentrations less than 90%, our previous studies under hyperoxia (≥90%) showed increased cardiomyocyte size and heart cross‐sectional area in aged males but decreased in aged females following hyperoxia exposure (Vichare et al., 2022). Other murine studies have reported similar sex‐dependent responses under hyperoxia, where males are more susceptible to oxidative stress, exhibiting elevated expression of antioxidative proteins such as Hmox1 and a higher GSH/GSSG ratio, indicating an increased reactive oxygen species (ROS) burden (ElBeck et al., 2024). This heightened oxidative stress in males may drive hypertrophy through activation of redox‐sensitive signaling pathways, including MAPK and NF‐κB, which promote cardiomyocyte growth (Giordano, 2005) and ultimately increase heart weight.

Our previous research has shown that lung edema occurs in all mice, regardless of age and gender, after 72 hours of hyperoxia (90% O_2_) (Vichare et al., 2022). In contrast, except for male end‐of‐life mice at 70% O_2_, none of the mice showed any significant lung edema in this study (Figure 1c). This implies the effects of higher O_2_ concentrations on the lungs do not take place until 70% O_2_ in male mice and only at hyperoxia (≥90% O_2_) in females. This is also reported in other studies where 60% O_2_ does not consistently induce pulmonary edema (Minkove et al., 2023). Convention states the lung is the first organ affected, and then other systemic effects occur (Amarelle et al., 2021). However, this is the first study to suggest earlier cardiac sensitivity compared to the lung, where supplemental oxygen at low concentrations can impact cardiac structure and function even before lung edema takes place. This lack of lung edema at lower O_2_ concentrations despite physical, electrical and functional remodeling of the heart suggests a high sensitivity of the heart towards oxygen concentrations to varying O_2_ levels. Therefore, monitoring cardiac physiology and function is more important in end‐of‐life, hospice or palliative care patients when subjected to supplemental oxygen than the lung or any other organs. Additionally, in this study we also report that in end‐of‐life mice male groups at all O_2_ concentrations, except 40% O_2_, showed significantly higher lung wet/dry ratio than their female counterparts, differing from previous data on young and aged mice, where no significant difference between sexes (Ayalasomayajula et al., 2024; Rodgers, Rodgers, et al., 2019; Saleem et al., 2023; Vichare et al., 2022).

### 4.2 Functional remodeling

In prior studies, we have reported that hyperoxia significantly increases %EF and %FS in both sexes and ages (Ayalasomayajula et al., 2024). Interestingly, our end‐of‐life mice show a significant decrease in %EF and %FS at all O_2_ concentrations in both sexes compared to normal air (Figure 2a–c), demonstrating that end‐of‐life mice behave uniquely under oxidative stress. This phenomenon is also seen in another murine model, where RV ejection fraction decreased in rats (both sexes) exposed to hyperoxia (FiO_2_ ≥85%) for the first 14 days of their birth and evaluated cardiac function after 1 year of age due to RV hypertrophy and mitochondrial dysfunction (Goss et al., 2017). Another study investigating the effect of reactive oxygen species and age on functional parameters found that young and middle‐aged mice of both sexes displayed preserved EF% and FS%, while the same parameters were significantly lower in old mice, revealing an age‐dependent decline of contractile function (Kang et al., 2023).

In addition to changes in these contractility indices, we have previously found that hyperoxia results in significant decreases in both SV and CO in young and aged mice, independent of sex (Ayalasomayajula et al., 2024). Similarly, end‐of‐life mice show a significant decrease in SV and CO at all O_2_ concentrations compared to normal air, albeit to a larger degree (Figure 2d,e). Similar observations were reported in human studies, where acute normobaric hyperoxia resulted in a 10‐15% decrease in SV and CO in both healthy volunteers and those with coronary artery disease (CAD) (Smit et al., 2018). Increased transcutaneous oxygen pressures and 100% FiO_2_ treatment in humans also produce significant decreases in SV, similar to the trends shown in our data (Bak et al., 2007; Harten et al., 2003). These decreases in CO and SV may be explained by coronary blood flow decreasing and coronary resistance increasing when the heart is subject to high concentrations of O_2_ (Farquhar et al., 2009). Overall, for the first time, in this study, we are reporting significant changes in cardiac function, even at low O_2_ concentrations, in end‐of‐life mice model suggesting CVD risk and reduced quality of life.

A prominent sex‐dependent divergence in response to supplemental oxygen was clearly reported in all our previous studies, including the current study. Female mice demonstrated significantly higher baseline systolic function (Figure 2) compared to males (FS%, EF%, SV, and CO) (Ayalasomayajula et al., 2024; Vichare et al., 2022). Several studies have noted that females display higher functional parameters (LVEF and FS) than male mice and have found this difference to become more pronounced with advancing age (ElBeck et al., 2024) (Zhang et al., 2021). Longitudinal assessments of %EF in mice from 3 to 16 months of age have shown that while %EF overlaps between sexes at younger ages, a substantial gap emerges by 16 months, with females demonstrating approximately a 20% higher %EF (Koch et al., 2013). These findings reinforce that aging amplifies sex differences in functional cardiac performance. Additional observation found in this study is that end‐of‐life female mice experience a significant decrease in systolic function and overall cardiac function at a larger magnitude than their male counterparts, specifically from 60% or higher O_2_ concentrations (Figure 2). These outcomes underscore sex as an imperative biological factor to consider when evaluating the possibility of O_2_ therapy and question the belief that oxygen treatment impacts all patients uniformly. Because of significant decline in functional parameters in end‐of‐life female, one factor that should be considered when thinking about its translational relevance is menopause in older women. Clinical studies suggest that the loss of estrogen in menopause plays a crucial role in worsening the risk for heart failure, increased cardiac tissue stiffness, and decreased functional performance (Sung et al., 2022). Clinical data has also found women with premature/early menopause have higher rates of CVD than those who experience menopause at 50‐51 years (Zhu et al., 2019). This suggests that menopause, along with sex, should be considered when considering oxygen therapy for patients in end‐of‐life cares.

### 4.3 Electrical remodeling

In this study, we observed a non‐uniform pattern in electrical disparities in end‐of‐life care mice, which also differs between males and females (Figure 3). Although previous reports from our lab showed a significant increase in QTc interval at just 24 h of hyperoxia exposure in both sexes and ages (Ayalasomayajula et al., 2024), this is the first study showing the impact of supplemental oxygen at lower concentrations. In this study, end‐of‐life care mice do not show any significant change in QTc interval until 60% or higher of oxygen concentrations, whereas only at 70% oxygen in females (Figure 3d). Prolonged QTc in animal and human models has been linked to the generation of cardiac arrhythmias that can progress to ventricular fibrillation and even sudden cardiac death, and thus the QTc increase observed in these end‐of‐life care mice exposed is best interpreted as a marker of elevated arrhythmic and mortality risk (Chapalamadugu et al., 2015; Varro & Baczko, 2011; Zhang et al., 2011).

Beyond the decline in functional parameters, sex affected the pattern and extent of changes in electrical parameters as well. End‐of‐life female mice showed an earlier and more significant extension of both QTc and QRS duration than males. Female groups experience significant increase in QRS and QTc starting from 60% O_2_, while males don't show increase until 70% O_2_ (Figure 3c,d). The sex differences observed in QRS interval are specific to end‐of‐life mice, as young and aged mice exposed to hyperoxia in previous experiments did not display any significant sex divergence (Ayalasomayajula et al., 2024; Vichare et al., 2022). Information on sex‐specific electrical reactions to oxidative stress is scarce. Previous reports established sex variations in baseline electrophysiological parameters, with women having a longer QTc (Vink et al., 2018), increased vulnerability to QT‐prolonging disturbances, and a greater occurrence of specific arrhythmias in women due to their higher QTc at baseline (Diez‐Escute et al., 2023). However, no studies have explored this sex influence as it plays a part in cardiac remodeling caused by oxidative stress specifically. Our data demonstrates the novel possibility that end‐of‐life females are more susceptible to electrophysiological changes at lower O_2_ concentrations than end‐of‐life males.

While our results clearly demonstrate oxygen‐dependent remodeling, a limitation is the lack of direct arterial blood gas measurements to correlate cardiac function with exact PaO_2_ levels. Furthermore, though food and water were provided ad libitum, the observed body weight decline (particularly in females at 70% O_2_) may be partially influenced by reduced intake during the exposure period, a phenomenon observed in our previous hyperoxia studies (Rodgers et al., 2021). Future studies should aim to differentiate the primary effects of oxidative stress from secondary metabolic changes.

## 5 CONCLUSION AND IMPLICATIONS

This research provides firsthand proof that supplemental oxygen consistently induces dose‐dependent cardiac remodeling in end‐of‐life mice, suggesting negative effects of supplemental oxygen on a population with increased vulnerability to oxidative stress. These changes involve impacts on functional parameters (significantly decreased FS%, EF%, SV, and CO) and electrophysiological parameters (lengthened QRS and QTc intervals), suggesting that supplemental oxygen, as low as 40% O_2_, acts as significant cardiac stressors instead of improving comfort and quality of life for end‐of‐life or palliative care patients. Importantly, this remodeling starts at low O_2_ concentrations and becomes even more pronounced at higher concentrations, implying use of supplemental oxygen in end‐of‐life care should be administered when necessary and with precaution. Our results also reveal a sex influence on supplemental oxygen‐triggered consequences for cardiac remodeling. While female myocardium in end‐of‐life forfeits its functional parameters at lower O_2_ concentrations than their male counterparts, the male group showed susceptibility to electrical remodeling at lower O_2_ concentrations than females. Clinical oxygen therapy protocols might also require consideration of sex and age‐related risks, suggesting more cautious oxygen goals or intensified surveillance for female and elderly patients, particularly those with preexisting cardiovascular conditions or additional risk factors, given the sex and age‐related susceptibility linked to cardiac remodeling. Additional measures should be taken with elderly patients in palliative care or hospice, and oxygen should not be provided only for the purposes of comfort. Unless supplemental oxygen is needed or necessary, it should not be administered as a standard of care, as it is shown to have serious cardiovascular effects in an end‐of‐life mouse model.

## AUTHOR CONTRIBUTIONS

**Rohan Cheruku:** Data curation; formal analysis; methodology; validation. **Drithi Chidanand:** Formal analysis; methodology; validation. **Sashank Rangarajan:** Formal analysis; methodology; validation. **Nishank Rangarajan:** Formal analysis; methodology; validation. **Siva Kumar Panguluri:** Conceptualization; data curation; formal analysis; investigation; methodology; project administration; resources; software; supervision; validation; visualization.

## FUNDING INFORMATION

The authors have nothing to report.

## CONFLICT OF INTEREST STATEMENT

The authors declare no conflicts of interest.

## Data Availability

All the data analyzed and presented in this study are available from the corresponding author on reasonable request and requests are processed according to institutional data sharing policies (https://www.usf.edu/data‐governance/data‐sharing.aspx).
